# Does Number of Ports Affect Outcomes in Patients Undergoing Laparoscopic Pyloromyotomy? Retrospective Chart-Review Study

**DOI:** 10.5402/2012/745964

**Published:** 2012-02-01

**Authors:** Tariq O. Abbas, Adel Ismail

**Affiliations:** Pediatric Surgery Department, Hamad General Hospital, P.O. Box 3050, Doha, Qatar

## Abstract

*Background*. Although open Ramstedt's pyloromyotomy is the gold standard for the surgical management of infantile hypertrophic pyloric stenosis, laparoscopic pyloromyotomy has been found highly successful. Various factors, however, can affect the outcomes of surgical interventions in these patients. We observed a relationship between the number of ports used and outcome in patients undergoing laparoscopic pyloromyotomies. *Methods*. We retrospectively assessed the medical records of selected group of patients who underwent laparoscopic pyloromyotomy in our institution. Factors analyzed included operation time, length of hospital stay, postoperative complications, and time to postoperative full feeding. *Results*. We observed failure of myotomy in both two patients who underwent laparoscopic pyloromyotomy using only two working ports compared to successful myotomies in the remaining patients. *Conclusion*. Laparoscopy provides good results in terms of intraoperative exposure and cosmesis. However, standardized surgical technique with two working ports is advisable, and this can trigger further research to be ascertained.

## 1. Introduction

Infantile hypertrophic pyloric stenosis (IHPS) is a common condition affecting young infants; despite its frequency, IHPS has been recognized as a condition for only a little over a century and its etiology remains unknown [1]. Among the approaches used for pyloromyotomy are umbilical skin-fold incision [2] and laparoscopy [3].

Several factors can affect the outcomes of surgical intervention in these patients. In this case series, we observed a relationship between the number of ports and the outcome in patients undergoing laparoscopic pyloromyotomy.

## 2. Methods

We assessed patients who had undergone laparoscopic pyloromyotomy at our institution. IHPS was diagnosed by pyloric muscle thickness >4 mm and length >14 mm on ultrasonography. The study protocol was approved by our institution's Internal Review Board. There was no external source of funding.

Patients were excluded if they had been born prematurely (before 37 weeks of gestation) or had recent respiratory infections, major developmental anomalies, or had undergone prior abdominal surgery.

All the procedures were performed by one surgeon. The case notes of these patients were retrospectively reviewed for age of the patients at operation, subsequent surgical interventions, and patient outcomes.

The primary end points of this study were rate of postoperative vomiting and ultimately the need for revision pyloromyotomy. Secondary outcome measures included operation time, length of hospital stay, postoperative complications, and follow-up findings.

## 3. Results

We assessed 7 children who fit the inclusion criteria mentioned above and underwent laparoscopic pyloromyotomy (Table 1). Only one of the patients has a positive family history of the disease.

However, there is no remarkable difference in the operative or anesthesia time between the two groups of patients who underwent laparoscopic pyloromyotomy using either two or three working ports. On the other hand, procedures performed using only two working ports resulted in failure of complete myotomy and both needed revision pyloromyotomy consequently. No postoperative wound infection or dehiscence was recorded.

## 4. Discussion 

The surgical procedure for pyloromyotomy was first described in 1912. Attention to detail is necessary to minimize morbidity and mortality [4]. When compared with open pyloromyotomy, the laparoscopic approach appears equally safe and effective, with superior cosmetic results [5]. A systematic review in 2009 found that the rate of total complications was significantly lower with the laparoscopic than the open approach, due primarily to a lower wound complication rate [6]. Moreover, the laparoscopic procedure is as quick as the open procedure, has low morbidity, is devoid of major wound-related problems, and can be easily taught [7]. 

An adequate pyloromyotomy must balance between the risk of perforation and the risk of incomplete myotomy, although an inability to palpate the divided pylorus makes the evaluation of these risks particularly challenging [8]. 

In one of the largest series to date, the incidence of incomplete pyloromyotomy was 4%, suggesting the need for conversion whenever the quality of the myotomy was reduced. In contrast, about 15% of patients in that trial experienced postoperative vomiting, with this factor not influenced by experience with laparoscopic pyloromyotomy [7]. 

**Figure 1:**
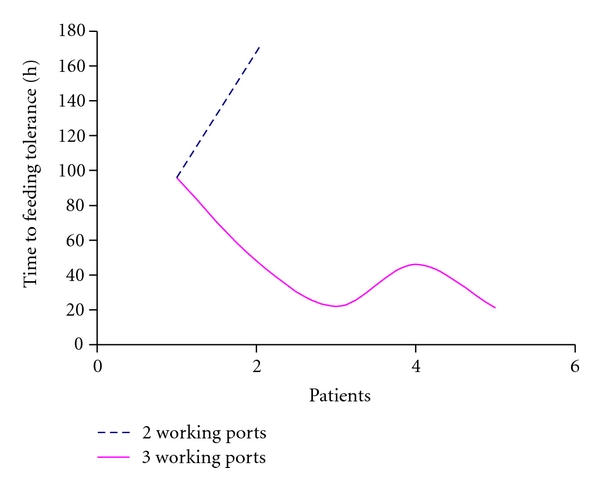


However, we observed a relationship between the number of ports and postoperative feeding tolerance in patients undergoing laparoscopic pyloromyotomies. A representative diagram of the time to postoperative feeding tolerance is shown in Figure 1. 

Procedures performed using only 2 working ports resulted in failure of complete myotomy, despite being able to visualize the bulging mucosa. This may have resulted from an oblique angle to the myotomy line, leading to the easing of both sides of the muscle and refusion.

Similar to previous findings, none of our patients experienced postoperative wound infection or dehiscence. By contrast, the incidence of these complications after open pyloromyotomy has been reported to be 6.7% [5, 9, 10].

In conclusion, laparoscopy provides good results in terms of intraoperative exposure and cosmesis; however, standardized surgical technique with two working ports is advisable. 

## Figures and Tables

**Table 1:** 

	Patient 1	Patient 2	Patient 3	Patient 4	Patient 5	Patient 6	Patient 7
Duration of symptoms (d)	2	15	3	7	7	10	4
Family history	−ve	−ve	−ve	−ve	−ve	−ve	+ve
Preoperative weight (kg)	3	4.5	4.6	4	4	3	5.4
Pylorus length (mm)	17 × 10	24 × 3	25 × 5	25 × 7	15 × 4	NA	18 × 19
Serum electrolytes (mmol/L) Before operation							
Chloride	132	104	87	84	85	87	104
Sodium	—	141	137	136	136	137	137
Bicarb.	28	30	29	32	38	22	—
Number of working ports	3	2	3	2	3	3	3
Conversion to open	−ve	−ve	−ve	−ve	−ve	−ve	−ve
Postoperative complications	−ve	−ve	−ve	−ve	−ve	−ve	−ve
Postoperative vomiting	−ve	+ve	−ve	+ve	−ve	+ve	−ve
Revision pyloromyotomy	−ve	+ve	−ve	+ve	−ve	−ve	−ve
Duration of surgery (min)	35	35	50	—	30	40	30
Duration of anesthesia (min)	65	55	85	80	50	50	65
Time to full feeding (h)	96	96	48	168	22	46	21
Postoperative length of hospital stay (d)	4	4.5	2.5	9	1	3	1
Wound infection	−ve	−ve	−ve	−ve	−ve	−ve	−ve
Wound dehiscence	−ve	−ve	−ve	−ve	−ve	−ve	−ve
